# Comparison of combined leflunomide and low-dose corticosteroid therapy with full-dose corticosteroid monotherapy for progressive IgA nephropathy

**DOI:** 10.18632/oncotarget.16468

**Published:** 2017-03-22

**Authors:** Lulin Min, Qin Wang, Liou Cao, Wenyan Zhou, Jiangzi Yuan, Minfang Zhang, Xiajing Che, Shan Mou, Wei Fang, Leyi Gu, Mingli Zhu, Ling Wang, Zanzhe Yu, Jiaqi Qian, Zhaohui Ni

**Affiliations:** ^1^ Department of Nephrology, Molecular Cell Lab for Kidney Disease, Ren Ji Hospital, School of Medicine, Shanghai Jiao Tong University, Shanghai, China

**Keywords:** IgA nephropathy, leflunomide, proteinuria, corticosteroids, renal survival

## Abstract

IgA nephropathy is the most common primary glomerulonephritis and one of the leading causes of end-stage renal disease. We performed a randomized, controlled, prospective, open-label trial to determine whether leflunomide combined with low-dose corticosteroid is safe and effective for the treatment of progressive IgA nephropathy, as compared to full-dose corticosteroid monotherapy. Biopsy-proved primary IgA nephropathy patients with an estimated glomerular filtration rate ≥ 30 ml/min/1.73m^2^ and proteinuria ≥1.0 g/24h were randomly assigned to receive leflunomide+low-dose corticosteroid (leflunomide group; n = 40) or full-dose corticosteroid (corticosteroids group; *n* = 45). The primary outcome was renal survival; secondary outcomes were proteinuria and adverse events. After 12 months of treatment and an average follow-up of 88 months, 11.1% *vs*. 7.5% of patients reached end-stage renal disease and 20% *versus* 10% of patients had a ≥ 50% increase in serum creatinine in the corticosteroids and leflunomide groups, respectively. Kaplan-Meier analysis did not reveal a between-group difference in these outcomes. Decreases in 24-hour proteinuria were similar in the two groups during the treatment period, but a more marked reduction was observed during follow-up in the leflunomide group. Although the incidence of adverse events was similar in the two groups, serious adverse events were observed only in the corticosteroid group. Thus, leflunomide combined with low-dose corticosteroid is at least as effective as corticosteroid alone for the treatment of progressive IgA nephropathy, and showed a greater reduction of proteinuria during long-term follow-up and fewer severe adverse events.

## INTRODUCTION

IgA nephropathy (IgAN) is the most common primary glomerulonephritis (GN) and one of the leading causes of end-stage renal disease (ESRD) worldwide [1–2]. The incidence of IgAN is particularly high in the East Asian population, accounting for > 40% of kidney biopsy specimens obtained from patients with primary GN in China or Japan [1]. IgAN is recognized as a chronically progressive disease. Among patients with biopsy-proven IgAN, 15%-20% reach ESRD within 10 years and 20%-40% within 20 years [3]. At present, treatment options for IgAN are still very limited. Disease management mainly consists of controlling blood pressure and lipid levels and reducing proteinuria by ACEI or ARB, together with other supportive treatment. In addition, the 2012 KDIGO GN Guidelines recommend that patients with persistent proteinuria (≥1 g/d and eGFR > 50 ml/min per 1.73m^2^) despite 3-6 months of optimized supportive care should receive a 6-month course of CS therapy [4]. However, the long-term use of CS is associated with many side effects, including abnormal glucose metabolism, osteoporosis, *etc*. Some patients with IgAN may also develop CS resistance or dependence, or relapse after withdrawal of steroids. Therefore, several clinical trials have been performed to determine whether combination therapy of steroids and immunosuppressive medications such as cyclophosphamide [5–6], azathioprine [7–8], mycophenolate mofetil [9–14], tacrolimus [15], and mizoribine [16] could provide better renal outcomes and fewer side effects. However, the results were inconsistent [17]. Leflunomide (LEF), an immunosuppressive agent that inhibits the synthesis of pyridines, has been widely used in rheumatoid and renal diseases in recent years [18–21]. Several randomized trials evaluated the efficacy of LEF in the treatment of IgAN. The results suggested that LEF reduced the amount of proteinuria and ameliorated renal function deterioration, with only mild side effects [22–24]. Yet, randomized long-term follow-up studies are lacking. Therefore, we conducted here a randomized, controlled, prospective, open-label trial with long-term follow-up to determine whether LEF plus low-dose CS is safe and effective for the treatment of progressive IgAN, as compared to full-dose CS alone.

## RESULTS

**Figure 1:**
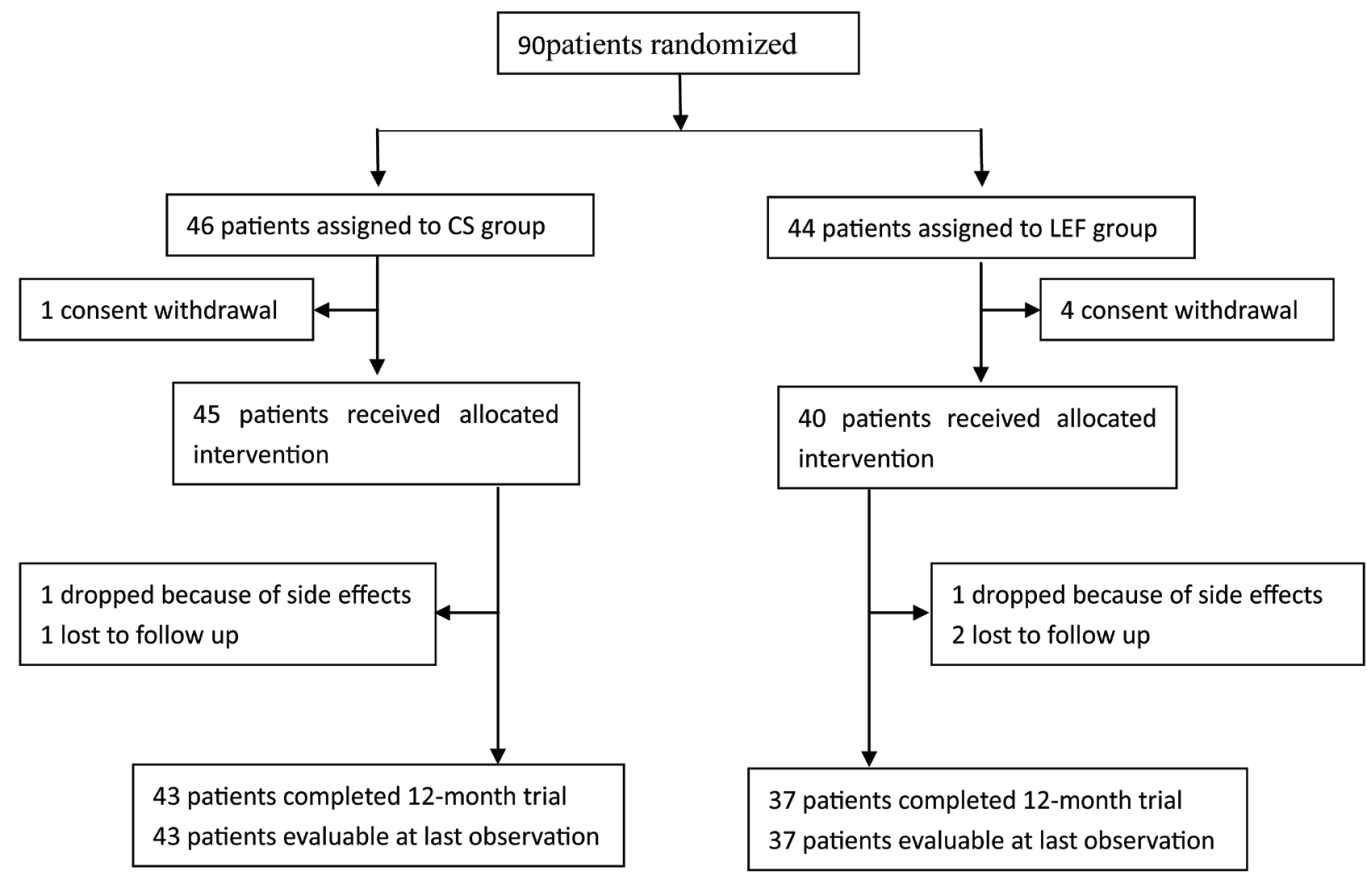
Patient enrollment and follow-up

In total, 90 patients with IgAN were randomly assigned to one of two groups: 1) CS (full-dose prednisone); 2) LEF (LEF plus low-dose prednisone). Five patients dropped off before treatment. Of the remaining 85 patients, 45 were assigned to the CS group and 40 to the LEF group. 80 patients (43 patients in the CS group and 37 patients in the LEF group) completed the 12-month treatment and were followed up for a median period of 88 months (Figure 1).

### Baseline characteristics

Table 1 shows the baseline characteristics of the groups. Age, sex, systolic blood pressure (SBP), diastolic blood pressure (DBP), 24-hour proteinuria, serum creatinine (SCr) levels, eGFR (by CKD-EPI equation), histological characteristics, and other pre-treatment clinical biochemical indexes were comparable between groups.

**Table 1:** Baseline clinical and histological characteristics of the study subjects

Characteristic	CS group (*n* = 45)	LEF group (*n* = 40)	*p* value
Age (years)	36.60 ± 11.53	36.90 ± 10.49	0.901
Sex (male)	22	14	0.272
SBP (mmHg)	121.69 ± 16.78	122.15 ± 12.98	0.889
DBP (mmHg)	79.29 ± 11.48	79.05 ± 9.86	0.919
SCr (μmol/L)	95.14 ± 31.55	92.43 ± 33.99	0.705
UA (μmol/L)	380.08 ± 87.44	356.77 ± 104.54	0.266
UPE (g/24h)	1.78 (1.31, 3.49)	1.91 (1.18, 2.88)	0.420
ALB (mmol/L)	37.73 ± 4.10	38.25 ± 4.13	0.569
GLU	5.00 ± 0.53	4.92 ± 0.57	0.493
Hb	133.89 ± 16.81	131.55 ± 18.12	0.542
ALT (U/L)	19.94 ± 11.07	24.21 ± 21.40	0.249
AST (U/L)	19.85 ± 5.86	22.02 ± 11.26	0.067
TG (mmol/L)	1.90 ± 0.93	1.97 ± 1.12	0.737
TC (mmol/L)	5.19 ± 1.09	5.10 ± 1.32	0.730
LDL (mmol/L)	3.16 ± 0.80	3.11 ± 0.96	0.799
eGFR (ml/min/1.73m^2^)	84.26 ± 29.05	84.10 ± 25.55	0.979
segmental or global glomerulosclerosis/ total glomeruli (%)	39.69 ± 20.79	40.13 ± 26.32	0.932
interstitial fibrosis and chronic inflammation (score)	1.58 ± 0.71	1.56 ± 0.67	0.919
tubule atrophy (score)	1.52 ± 0.67	1.51 ± 0.71	0.972
inflammatory cell infiltration (score)	2 (1, 2)	1 (1, 2.88)	0.555
crescentic glomeruli/ total glomeruli (%)	4 (0, 11.5)	5 (0, 10)	0.859
Follow-up (months)	89.12 ± 22.61	87.22 ± 21.24	0.701

Values are presented as means ± standard deviation (SD) if the variables showed a normal distribution and as medians (IQR) if the variables did not show a normal distribution; n (%) was used for the categorical variables.

SBP: systolic blood pressure; DBP: diastolic blood pressure; SCr: serum creatinine; UA: serum uric acid; UPE: Urine protein excretion; ALB: serum albumin; GLU: glucose; Hb: hemoglobin; ALT: alanine transaminase; TG: triglyceride; TC: cholesterol; LDL: low density lipoprotein; eGFR: estimated glomerular filtration rate; AST: aspartate aminotransferase.

### Outcome of treatment

After 12 months of treatment, proteinuria (g/24h) levels decreased significantly in both groups. In the CS group, it decreased from 2.16 (IQR 1.36 to 3.5) at baseline to 0.83 (IQR 0.32 to 1.94) at 6 months, and to 0.65 (IQR 0.17 to 1.46) at 12 months (*p* < 0.01). In the LEF group, proteinuria decreased from 1.94 (IQR 1.21 to 2.87) at baseline to 0.54 (IQR 0.15 to 1.15) at 6 months, and to 0.29 (IQR 0.08 to 0.69) at 12 months (*p* < 0.01). Whereas baseline and 6-month values were similar between groups, 12-month proteinuria levels in the LEF group were significantly lower than in the CS group (*p* < 0.05; Table 2).

**Table 2:** Outcomes of Treatment

		CS group (*n* = 45)	LEF group (*n* = 40)	*p* value
UPE (g/24h)	baseline	2.16 (1.36, 3.5)	1.94 (1.21, 2.87)	0.372
	Month 6	0.83 (0.32, 1.94)**	0.54 (0.15, 1.15)**	0.070
	Month 12	0.65 (0.17, 1.46)**	0.29 (0.08, 0.69)**	0.022
SCr (μmol/L)	baseline	95.40 ± 32.27	92.59 ± 35.02	0.712
	Month 6	94.46 ± 35.31	87.75 ± 32.06	0.379
	Month 12	96.24 ± 38.53	87.66 ± 32.87	0.292
eGFR (ml/min/1.73 m^2^)	baseline	83.64 ± 29.51	84.17 ± 26.03	0.933
	Month 6	85.32 ± 30.80	87.79 ± 26.01	0.702
	Month 12	83.74 ± 31.54	87.51 ± 27.66	0.575

*P < 0.05 *versus* baseline value

**P < 0.01 *versus* baseline value

UPE: urine protein excretion

Scr: serum creatinine

eGFR: estimated glomerular filtration rate

Renal function was stable in both groups through the 12-month treatment period, as indicated by SCr and eGFR, which were no different between groups (Table 2).

At the end of treatment, a total of 31 patients (68.9%) achieved remission (14 CR, 17 PR) in the CS group, whereas a total of 27 patients (67.5%) achieved remission (16 CR, 11 PR) in the LEF group. However, among the remission patients, 5 patients in the CS group and 4 in the LEF group experienced relapse within one year after withdrawal of drugs. There was no difference in the total remission and relapse rates between the two groups.

### Renal survival

**Figure 2:**
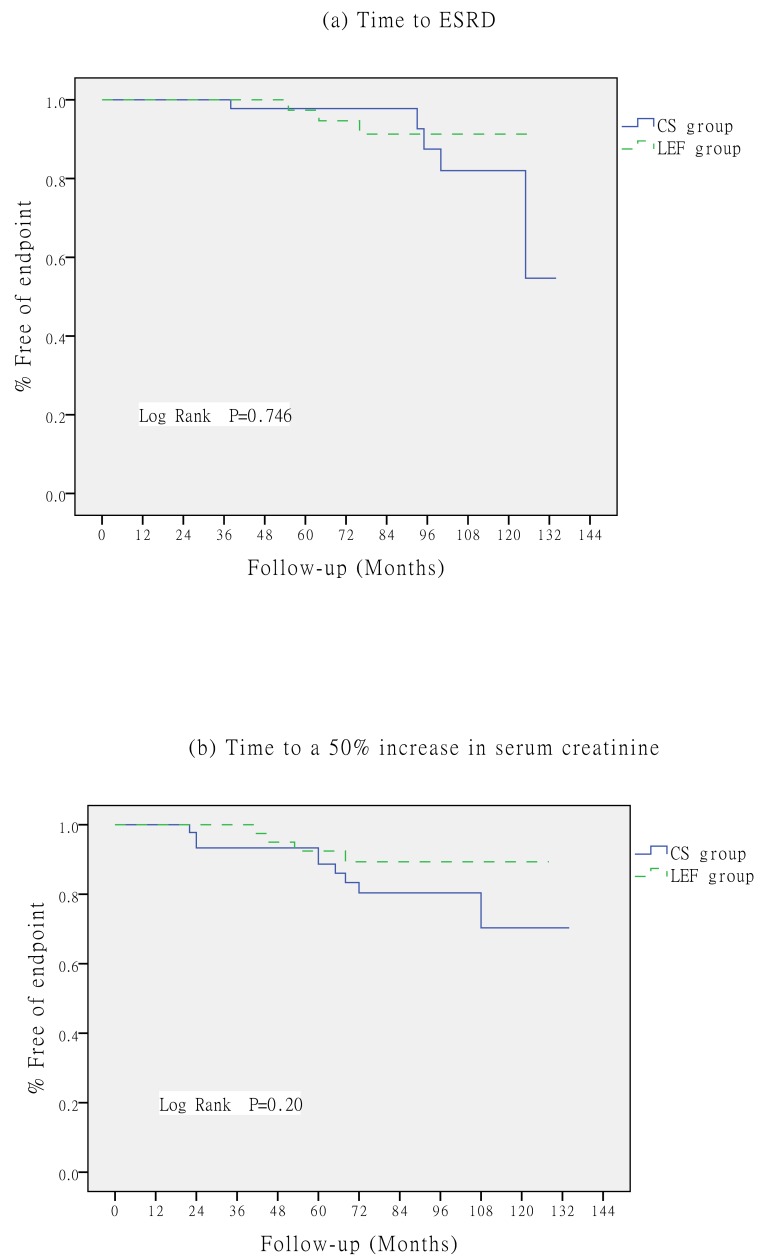
Kaplan-Meier survival for primary outcomes; Log rank significance for ESRD a. and for a 50% increase in SCr b

No statistically significant differences in primary outcomes were noted between groups. However, there was a trend towards better outcomes in the LEF group. The rate of ESRD was 11.1% (5/45) in the CS group *vs*. 7.5% (3/40) in the LEF group. A 50% increase in SCr from baseline occurred in 20% of patients (9/45) in the CS group *vs*. 10% of patients (4/40) in the LEF group. The cumulative probabilities of event-free survival for the primary outcomes were calculated using the Kaplan-Meier method. No significant between-treatment differences were observed (Figure 2).

### Proteinuria

**Figure 3:**
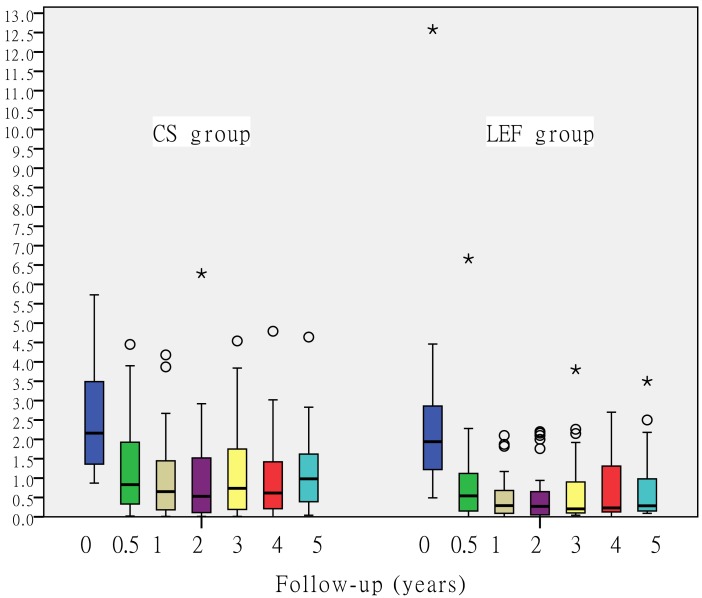
Changes in 24-hour proteinuria over time In both groups the proteinuria decreased significantly from baseline and no rebound was observed during the follow-up period. (Significantly lower urine protein excretion rates were observed at 24, 36, and 60 months in the LEF group) Significantly lower urine protein excretion rates were observed at 24, 36, and 60 months in the LEF group (*P* < 0.05; Wilcoxon-Mann-Whitney test). The lines crossing the boxes indicate the median; boxes indicate the IQR, i,e. the spread of the middle 50% of the values; the whiskers show the largest and smallest observed values that are < 1.5 box lengths from the 25th or 75th percentile. Circles and asterisks indicate more extreme values.

The results summarized in Table 2 show, as described above, that 24-hour proteinuria levels decreased significantly in both groups during the 12-month treatment period. After withdrawal of drugs, there was no rebound in either group during the follow-up period (Figure 3). The 24-hour proteinuria levels in the CS group were 0.53 (IQR 0.11 to 1.32) at 24 months, 0.61 (IQR 0.18 to 1.68) at 36 months, 0.62 (IQR 0.21 to 1.48) at 48 months, and 0.96 (IQR 0.32 to 1.75) at 60 months. Meanwhile, in the LEF group proteinuria values were 0.16 (IQR 0.04 to 0.58) at 24 months, 0.2 (IQR 0.1 to 0.95) at 36 months, 0.23 (IQR 0.13 to 1.31) at 48 months, and 0.27 (IQR 0.15 to 1.03) at 60 months. Significantly lower urine protein excretion rates were observed at 24, 36, and 60 months in the LEF group. (*P* < 0.05; Figure 3).

### Blood pressure control and RAS blockade

During the follow-up period, the patients’ blood pressures were stable and similar between the two groups (SBP = 121.91 ± 15.03 mmHg for CS *vs*. 121.51 ± 14.58 mmHg for LEF, *p* = 0.78; DBP = 79.18 ± 10.69 mmHg for CS *vs*. 78.34 ± 9.45 mmHg for LEF, *p* = 0.43). Thirty-two patients (71.1%) in the CS group and 30 patients (75%) in the LEF group were treated with ACEI/ARB at the study entry. Three patients in the CS group and 2 in the LEF group received combined antihypertensive regimens at the beginning of the study. During the follow-up period, 6 patients in the CS group and 3 in the LEF group were given other antihypertensive drugs because their blood pressure did not achieve target levels.

### Compliance and adverse events

Table 3 lists the adverse events that occurred during treatments. Five of the 85 patients did not complete the 12-month therapy; 3 of them (1 in the CS group and 2 in the LEF group) were lost to follow-up and the other 2 (1 in each group) had serious pulmonary infection.

**Table 3:** Adverse events during the treatment period

	LEF group (*n* = 40)	CS group (*n* = 45)
Hepatotoxicity	3	2
Upper respiratory infection	4	4
Pulmonary infection	2	1
Diarrhea	1	0
Herpes-zoster virus infection	0	2
Pruritus	1	0
Insomnia	0	2
Alopecia	1	0
Abnormal glucose metabolism	0	2
Total (n)	12	13

In the LEF group, 12 of 40 patients (30%) suffered from adverse events including hepatotoxicity (*n* = 3), upper respiratory infection (*n* = 4), pulmonary infection (*n* = 2), diarrhea (*n* = 1), pruritus (*n* = 1), and alopecia (*n* = 1).

In the CS group, 13 of 45 patients (28.9%) suffered from adverse events including hepatotoxicity (*n* = 2), upper respiratory infection (*n* = 4), and pulmonary infection (*n* = 1). Other adverse effects such as herpes-zoster virus infection (*n* = 2), insomnia (*n* = 2), and abnormal glucose metabolism (*n* = 2) were only observed in the CS group.

No patients died during the follow-up. In the CS group, two serious adverse events were reported: one patient developed cerebral hemorrhage and diabetes, and another developed aortic dissection. No serious adverse events occurred in the LEF group.

## DISCUSSION

As treatment options for IgAN, a leading cause of primary glomerulonephritis, remain limited, there is a pressing need for improved therapies to alleviate this condition. The present results suggest that LEF plus low-dose CS is at least as effective as full-dose steroid monotherapy for the treatment of progressive IgAN, and is associated with both greater reduction of proteinuria during long-term follow up (12-88 months) and the absence of severe adverse events.

Consistent with our findings, previous investigations revealed that LEF could attenuate inflammation and ameliorate kidney injury [25–26]. Although some of the data on the effects of combined immunosuppressive medications plus steroids, as compared to steroids alone, on the remission rate of proteinuria are inconsistent [17] [30], several small clinical trials suggested that LEF can reduce proteinuria and delay IgAN progression [22–24]. A recent meta-analysis [27] of 13 RCTs involving 623 patients compared LEF (alone or plus steroid) with steroid therapy alone. LEF demonstrated a marked advantage on CR/PR of proteinuria, as compared with steroid therapy alone (CR/PR; RR, 2.64; 95% CI, 1.80-3.86; *P* < 0.00001). Although in our study the remission rate was similar between the LEF and CS groups, proteinuria levels were markedly lower in the LEF group during the follow-up period. On the other hand, the relapse rates in our trial, i.e. 16.1% in the CS group and 14.8% in the LEF group, were also similar between groups and comparable to those reported by Yuan et al. [28].

We observed no statistical differences regarding renal survival between the two groups. Consistent with our study, Pozzi et al. [8] added low-dose azathioprine to CS for IgAN treatment and found that the 5-year renal survival rate was no different than that achieved with steroids alone [29]. However, Tang et al. [11] reported that the 6-year renal survival with mycophenolate mofetil treatment was higher than that with conventional treatment with CS. These studies suggest that the use of immunosuppressive medications alone or in combination with CS for the treatment of IgA nephropathy results in equal or better long-term outcomes than conventional steroid monotherapy.

Most combination therapies with immunosuppressive medications cause considerable side effects. The adverse events of LEF in IgAN patients were summarized in a meta-analysis [27]. It showed that 4.7% of patients demonstrated elevated liver enzymes, 3.7% of patients exhibited digestive symptoms, and 4.4% patients had alopecia. In our study, the incidence of adverse events was similar between the CS and LEF groups. However, two severe adverse events occurred in the CS group during the follow-up period. Cerebral hemorrhage occurred in a 63-year-old male patient who developed abnormal glucose metabolism in the first round of prednisone and whose blood pressure control was not ideal. Aortic dissection occurred in a 35-year-old male patient with normal blood pressure. These two patients had a history of more than two cycles of CS therapy during the entire treatment and follow-up period, and it is possible that these adverse events were associated with long-term CS use. Our results suggest that LEF combined with low-dose CS is probably safer than full-dose steroid course for the treatment of IgAN, especially in those patients who could not tolerate full therapeutic CS dosages.

There were several limitations in our study. This is a single-center study with a relatively small sample size. Therefore, we could not perform subgroup analysis and true differences in renal survival between the groups might thus be masked. In addition, we could not perform intention-to-treat analysis, which is based on the initial treatment assignment and not on the treatment eventually received, because we could not access the clinical data of 5 patients who were withdrawn from the study. Finally, we did not have a detailed record of the frequency and treatment regimens of the disease relapse cases during the 88-month follow-up period.

In conclusion, a 12-month course of LEF combined with low-dose CS seems to be at least as effective as full-dose CS for the treatment of progressive IgAN, and is associated with both greater reduction of proteinuria and fewer severe adverse events during long-term follow-up. This regimen could be an alternative treatment option for IgAN patients with relative contraindications to full-dose corticosteroid therapy.

## MATERIALS AND METHODS

### Patients

This randomized, controlled, prospective, open-label, single-center trial enrolled patients from June 1, 2004 to June 30, 2010 at Ren Ji Hospital in Shanghai, China. All patients had biopsy-proven primary IgAN with renal biopsy samples examined independently by two pathologists. Patients aged 18-65 years were included if they had proteinuria ≥1.0 g/24h and an estimated glomerular filtration rate (eGFR) ≥30 ml/min/1.73m^2^ (calculated by CKD-EPI equation). Patients with any of the following conditions were excluded: (i) rapidly progressive IgA nephropathy (IgAN with rapid decline in renal function characterized histologically by necrotizing capillaritis or > 50% active crescents on biopsy) (ii) secondary IgA nephropathy due to systemic diseases such as Henoch-Schönlein purpura nephritis, hepatitis-associated nephritis, lupus nephritis, etc.; (iii) use of CS or other immunosuppressive agents within 6 months prior to randomization; (iv) serum creatinine (Scr) > 250umol/L; (v) severe infections; (vi) hepatitis B virus carriers and other chronic liver diseases; (vii) presence of malignancy, HIV infection, or acute central nervous system diseases; (viii) abnormal glucose metabolism; (ix) pregnancy or lactation; (x) poor compliance or allergy to study drugs. The study was reviewed and approved by the Ethics Committee at Ren Ji Hospital of Shanghai Jiao tong University Medical School (2002HL0133) and informed consent was obtained from all the participants.

### Treatment regimen and evaluations

**Figure 4:**
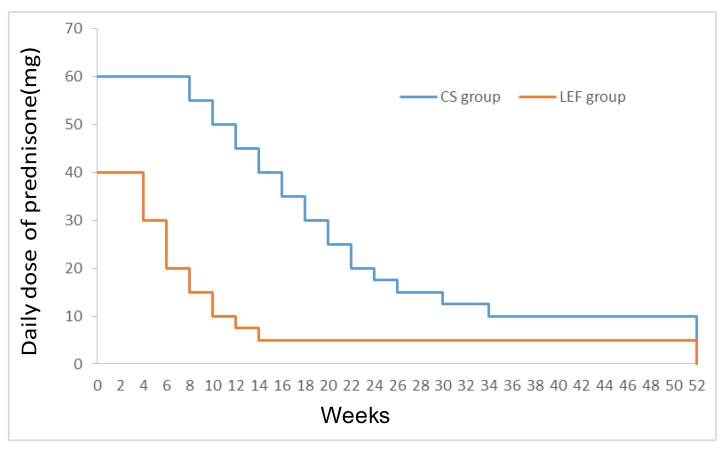
Plot of daily dose of prednisone-time profiles during 52-weeks follow-up for the CS and LEF groups CS group = full-dose corticosteroids alone; LEF group = leflunomide combined with low-dose of corticosteroids.

Patients were randomized to either leflunomide plus low-dose corticosteroid (LEF group) or to full-dose corticosteroid alone (CS group). Patients in the LEF group were treated with leflunomide 40 mg per day for 3 days, after which the dose was reduced to 20 mg per day and administered for 12 months, in conjunction with oral prednisone, 0.8 mg/kg/day for 4-6 weeks. The maximum daily dose of prednisone was 40mg. Then, prednisone was gradually tapered by 10, 5, and 2.5 mg to a maintenance dose of 5 mg per day. Patients in the CS group were treated with oral prednisone 1.0 mg/kg/day for 8-12 weeks, with a maximum daily dose of 60 mg. Then, the daily dose was tapered by 5 and 2.5 mg to a maintenance dose of 10 mg per day. The steroid regimen is illustrated in Figure 4.

No other immunosuppressive or cytotoxic agents were used. All patients were given ACEI/ARB unless blood pressure was ≤ 90/60 mmHg or if there was hypotension. Additional anti-hypertensive medications were also prescribed if blood pressure was > 130/80 mmHg with ACEI/ARB alone. Patients who developed hyperlipidemia, infection, or a hypercoagulable state were treated accordingly.

The duration of the treatment was 12 months. We evaluated clinical efficacy and monitored adverse events at 6- and 12-month time points. After one year of treatment, we continued to follow the patients for a median period of 88 months. We recorded the patients’ medications, clinical characteristics, and laboratory results at each follow-up visit. Urinary protein excretion was measured in 24-hour samples obtained at least once a year. During the follow-up period, the patients were retreated with the original regimen if the disease relapsed.

The criteria for the discontinuation of treatment included severe infection or other serious adverse reactions associated with the tested drugs, pregnancy, and death or lost to follow-up.

The primary outcome was ESRD or a 50% increase in baseline serum creatinine (SCr). The secondary outcome was changes in proteinuria over time.

Complete remission (CR) was defined as a urine protein excretion (UPE) < 0.3 g/d with stable Scr (defined as a change in SCr of 15% or less above baseline values). Partial remission (PR) was defined as having at least a 50% reduction in UPE compared with baseline or 0.3 g/d ≤ UPE < 3.5 g/d with stable Scr. No response (NR) was defined as a UPE > 3.5 g/d, or a < 50% reduction in UPE with or without renal deterioration [31]. Relapse was defined as the reappearance of significant proteinuria, defined as > 1.0 g/d and as a UPE increase of > 50% from the lowest level of proteinuria after remission [31–32].

### Renal biopsy

The following histopathological criteria were recorded: 1) percentage of segmental or global glomerulosclerosis/total glomeruli, 2) percentage of crescentic glomeruli/ total glomeruli, 3) extent (percentage of the biopsied area) of inflammatory cell infiltration (0 = absent, 1 = 1 to 24%, 2 = 25 to 50%, 3 = more than 50%), 4) extent (percentage of the biopsied area) of interstitial fibrosis and chronic inflammation (0 = no fibrosis or inflammation, 1 = 1 to 24%, 2 = 25 to 50%, 3 = more than 50%), 5) extent of tubular atrophy (percentage of atrophic tubules; 0 = no atrophy, 1 = 1 to 24%, 2 = 25 to 50%, 3 = more than 50%).

### Statistical analysis

Normally distributed variables were expressed as means ± SD and compared using a *t*-test or analysis of variance (ANOVA) as required. Non-parametric variables were expressed as median with range and compared using either the Mann-Whitney U test or the Kruskal-Wallis test. The chi-square test was employed for the categorical variables. Kaplan-Meier survival functions with the log-rank test were used to calculate the cumulative rates of the primary outcomes in the two groups. All tests were two-tailed, with *p*-values < 0.05 considered statistically significant. All statistics were done using SPSS 19.0.

## Abbreviations

ACEI: angiotensin-converting enzyme inhibitor
ALB: serum albumin
ALT: alanine transaminase
ARB: angiotensin receptor blocker
BUN: blood urea nitrogen
AST: aspartate aminotransferase
CKD: chronic kidney disease
CR: complete remission
CS: corticosteroids
DBP: diastolic blood pressure
eGFR: estimated glomerular filtration rate
ESRD: end-stage renal disease
GLU: glucose
GN: glomerulonephritis
Hb: hemoglobin
IgAN: IgA nephropathy
IQR: interquartile range
LDL: low density lipoprotein
LEF: leflunomide
NR: no response
PR: partial remission
SBP: systolic blood pressure
SD: standard deviation
SCr: serum creatinine
TC: total cholesterol
TG: triglycerides
UA: serum uric acid
UPE: 24-hour urinary protein excretion.
